# Negative Pragmatic Transfer in Bilinguals: Cross-Linguistic Influence in the Acquisition of Quantifiers

**DOI:** 10.1007/s10936-024-10101-9

**Published:** 2024-08-20

**Authors:** Greta Mazzaggio, Penka Stateva

**Affiliations:** 1 Department of Humanities, University of Florence, Via della Pergola 60, 50121 Firenze, FI Italy; 2 Center for Cognitive Science of Language, University of Nova Gorica, Vipavska 13, SI-5000 Nova Gorica, Slovenia

**Keywords:** Quantification, Cross-linguistic differences, Pragmatics, Semantics, Negative transfer

## Abstract

Building on the cross-linguistic variability in the meaning of vague quantifiers, this study explores the potential for negative transfer in Italian-Slovenian bilinguals concerning the use of quantificational determiners, specifically the translational equivalents of the English “many”, that is the Slovenian "precej" and "veliko". The aim is to identify relevant aspects of pragmatic knowledge for cross-linguistic influence. The study presents the results of a sentence-picture verification task in which Slovenian native speakers and Italian-Slovenian bilinguals evaluated sentences of the form "Quantifier X are Y" in relation to visual contexts. The results suggest that Italian learners of Slovenian, unlike Slovenian native speakers, fail to distinguish between "precej" and "veliko". This finding aligns with the negative transfer hypothesis. The study highlights the potential role of pragmatic knowledge in cross-linguistic transfer, particularly in the context of vague quantifiers.

## Introduction

Bilingualism and multilingualism are important societal challenges in the globalized world, in which the majority of people are capable of speaking more than one language (Grosjean, 2010), and understanding how bilinguals learn and use language is of key importance. While bilinguals have the advantage of being able to communicate in two or more languages, the acquisition and use of these languages can be complex (Luk & Bialystok, 2013) and subject to transfer effects (Dechert & Raupach, 1989; Treffers-Daller, 2009). The word “transfer” refers to the influence of one language on the learning or use of another language, which can be both positive and negative (Bardovi‐Harlig & Sprouse, 2018; Cummins, 1979). For instance, cross-linguistic transfer may result in increased vocabulary and syntactic knowledge in bilingual children, as they may transfer knowledge from one language to another (Bialystok, 2001). However, transfer effects can also result in a negative outcome, where bilinguals may transfer features of one language to another, leading to errors in language production (a.o., Odlin, 1989). Research has shown that bilingualism can have cognitive benefits, such as increased executive function and metalinguistic awareness (a.o., Bialystok, 2009; although this issue is still debated, see Van den Noort et al., 2019; Ware et al., 2020), but effective language teaching and learning requires a nuanced understanding of the complexity of bilingualism and the role of transfer effects in language acquisition and use.

The phenomenon of cross-linguistic influence in bilingual language acquisition (BLA) has been well-documented and is expected under the *Unitary-Language System Hypothesis* (Swain, 1972; Volterra & Taeschener, 1978), which posits that BLA involves the production of a hybrid grammatical system that reflects the influence of one language on the other. However, even under the alternative *Differentiated Language System Hypothesis* (Goodz, 1989; De Houwer, 1990), which suggests that bilingual children create independent grammatical systems for each language, language transfer can still be predicted by factors such as language distance, input/language dominance, and age (Müller & Hulk, 2001; Nicoladis, 2006; Serratrice et al., 2009, a.o.).

Research on negative transfer typically focuses on language acquisition's phonological (Dodd et al., 1996; Goldstein & Bunta, 2012; Goldstein & Washington, 2001) and morphosyntactic aspects (Sorace & Filiaci, 2006; Sorace & Serratrice, 2009; Tsimpli & Sorace, 2006). Additionally, research has addressed lexical transfer (Jarvis & Pavlenko, 2008; Pavlenko, 2009), where bilinguals may transfer word meanings and semantic properties from their first language (L1) to their second language (L2). Transfer in the pragmatic domain has received less attention in comparison to other areas of language acquisition.

Pragmatic transfer refers to the influence of a bilingual's L1 on the acquisition and use of pragmatic features in their L2. Pragmatic features include aspects of language use such as intonation, politeness, figurative language, and the use of some quantifiers as triggers for pragmatic inferences. This latter has been widely investigated within an L2-processing perspective (a.o., Dupuy et al., 2018; Mazzaggio et al., 2021; Slabakova, 2010), but rarely with a cross-linguistic perspective and considering transfer phenomena. This lack of research is likely due to the belief that the enrichment of meaning in pragmatics is governed by general principles that are not affected by cross-linguistic variations. However, it has yet to be proposed or argued in the literature that the process of inference derivation, which is central to pragmatic enrichment, can be language-specific, and that triggers for this process may differ in formal properties across languages.

## Cross-Linguistic Properties in the Acquisition of Quantifiers

The way quantities are realized can vary depending on the context or language used (Katsos et al., 2016; Stateva et al., 2019) and it is an interesting testing ground for negative transfer theories. Different languages have a diverse set of expressions for number words and expressions like "some", "many" and "a lot", as well as distinct grammatical structures for expressing quantities, for example, plural forms of nouns or quantifiers.

The meaning of "many" has garnered significant attention in the field of formal semantics. It has been recognized as having two primary uses: cardinal usage and proportional usage (Partee, 1988). In the context of the word "many", cardinal usage refers to a specific numerical value, whereas proportional usage refers to the proportion of objects in relation to a whole. For example, under the cardinal usage, "I have many books", would be appropriate to express a quantity of books which exceeds a contextually defined standard for owned books. But if someone says "I have many books" intending the proportional meaning, the sentence interpretation will be evaluated with relation to a contextually defined standard regarding a proportion of owned books from a particular well-defined finite set. Both the cardinal and proportional interpretations of "many" are vague, as they do not specify an exact quantity. This distinction between cardinal and proportional usages is important to understand the meaning of the word "many" and how it is used in different situations (Barwise & Cooper, 1981).

Degree-based semantic theories relate to a type of formal semantics that deals specifically with the meaning of vague predicates like “tall”, vague quantifiers involving determiners like “many”, “few”, “some”, degree operators like “-er”, “-est”, “too”, etc. Within most of the current theories, the meaning of the quantificational determiners is defined in terms of degrees.^1^ Degrees can be represented as points on a dense numerical scale, while the vague quantificational elements are thought of as modifiers of predicates (sets of individuals) in view of set cardinality (Cresswell, 1976; Hackl, 2000; Rett, 2018; Solt, 2009, 2015, a.o.). For example, the determiner "many" in a sentence like “Many dots are red” might be thought of as a relation between (i) the contextually provided standard (degree) that divides the number scale into a positive and a negative extension corresponding to cardinalities that count as involving *many* objects and cardinalities that count as involving *few* objects, respectively and (ii) the set of degrees *I* that make true the proposition that there are at least d-many individuals that are both dots and red, i.e. a relation between the degree of "many-ness" and the set of degrees that are either equal to or smaller than the degree that corresponds to the cardinality of the set of objects modified by "many" in the predicate (Rett, 2018; Solt, 2015).^2^ Under this approach, statements involving vague quantifiers can be evaluated using operations on the degrees of the scale, and the truth values of such statements are determined by comparing relevant interval (set of degrees) sizes on the scale (see Krasikova, 2011; Rett, 2008, 2018; Solt, 2015; Stateva & Stepanov, 2017).

Building on research that establishes a cross-linguistic variation between two languages with respect to their semantic representation of the vague quantifier “many”, Slovenian (Stateva & Stepanov, 2017) and Italian (Mazzaggio & Stateva, 2023), this work aims to investigate the L2 semantic/pragmatic processing of the pair of translational equivalents of the quantifier "many" in Slovenian by Italian L2 learners of Slovenian. We hypothesize that cross-linguistic variation feeds potential negative transfer.

It has been argued that languages like Russian (Babko-Malaya, 1998; Krasikova, 2011) can feature more than one lexical item like “many” but these are specified with respect to their semantics, and more specifically, they are specified as cardinal or proportional “many”. In contrast to Russian, Slovenian, as argued in Stateva and Stepanov (2017), has two translational equivalents of "many" (*precej* and *veliko*) but they express the same range of cardinal and proportional meanings, so they cannot be argued to specify different semantic content. However, there usually is a justification for the co-existence of two similar words within a single language (Clark, 1983); for this reason, Stateva and Stepanov conducted a series of sentence-picture evaluation tasks in addition to the theoretical investigations in the semantic makeup of both determiners and demonstrated how the interpretative differences between the two Slovenian m(any)-words (henceforth, m-words) can be explained through a pragmatic analysis. This analysis maintains semantic equivalence among the two lexical items, i.e. argues that they do not differ in their literal meaning of expressing a degree of “many-ness” but each m-word can be strengthened with an R/I-implicature or M-implicature, respectively (resulting from the application of the R, I or M-pragmatic principles).^3^ Essentially, Stateva and Stepanov extended Krifka’s (2007) and Horn’s (1989) proposals for deriving the interpretative differences between pairs of synonymous negative and positive degree predicates (for ex. < “unhappy”, “not happy” > ; < ”not unhappy”, “happy” >) through pragmatic strengthening. The main idea in the Horn/Krifka analysis of degree predicates which are related to a single scale is that given a standard degree that divides the scale into a positive and negative extension, synonymous predicates differentiate their interpretation pragmatically. For example, inside the positive extension reserved for < “not unhappy”, “happy” > , one item of the pair is assigned to the interval which is furthest from the standard, and thus strengthens its meaning to the stereotypical degree of the property ("happy”) through an I/R-implicature, while the other item, “not unhappy”, is associated with the remaining set of degrees which are positive but closer to the negative degrees of happiness in this case, and is strengthened with an M-implicature. Having argued for a degree analysis of the two Slovenian m-words, Stateva and Stepanov set the stage for applying the Krifka/Horn analysis to them and thus explaining why in many contexts the two m-words are interchangeable while on others, there are subtle interpretative differences which result in “veliko” being associated with higher numerical values than “precej”. In theoretical terms, the higher numerical values for “veliko” result from the stereotypical interpretation due to the I/R-implicature, while the lower ones for “precej” are due to the M-implicature which complements the otherwise identical lexical meaning. The study involved three manipulations in which native speakers of Slovenian evaluated sentences containing “veliko” and “precej”, either with or without the presence of the other alternative. When “precej” and “veliko” were not presented as overt alternatives of each other for describing visual contexts, participants assigned identical numerical bounds of the intervals corresponding to both lexical items. However, when “precej”- and “veliko”- sentences were presented as explicit linguistic alternatives within trials, participants distinguished their meaning by assigning a higher position on the numerical scale for “veliko”, and a lower one for “precej”. The results of the study supported Stateva and Stepanov’s proposal that the meanings of “precej” and “veliko” are pragmatically strengthened when appropriate contextual conditions are present. When both alternatives are considered, “precej” is linked to lower numerical values, which by hypothesis correspond adding a non-stereotypical implicature to the semantic interpretation, while “veliko” is linked to higher numerical values, as a result of strengthening the semantic interpretation with a stereotypical implicature.

A similar study has been recently applied to Italian language, obtaining different results (Mazzaggio & Stateva, 2023). Italian has at least two equivalents of the English "many"^4^: *molto* and *tanto* which similarly to Slovenian can be used to express both a cardinal and proportional reading in view of which the authors hypothesize that they can be distinguished pragmatically in a manner similar to the Slovenian “precej” and “veliko”. The distribution of the Italian m-words varies on their morphological realization such as adjective, adverb, or pronoun. Despite their informal intuition shared in the debriefing stage of the experiment that “tanto” refers to bigger quantities compared to “molto”, Italian native speakers did not evaluate “molto” and “tanto” differently when presented as alternatives, which contrasts with the results of the Slovenian speakers (Mazzaggio & Stateva, 2023; Montalto et al., 2010).^5^ These results suggest that the Italian "many" words, “molto” and “tanto”, have the same numerical bounds and are therefore perceived as interchangeable when used as amount modifiers. The analysis proposed by Mazzaggio and Stateva (2023) is that “molto” and “tanto” do not share the same semantic properties, with only “molto” being analyzed as a degree determiner, i.e. only “molto” is associated with the introduction of a degree scale while “tanto” is a quantifier over individuals. In other words, the effect of coinciding numerical bounds is epiphenomenal and results from the conceptual relation between sets of individuals that could be related through a quantificational determiner, on the one hand, and the scale of degrees expressing numerosity, on the other. This state of affairs may explain why as a pair of “synonyms” the two Italian m-words are not subject to pragmatic strengthening: they do not trigger inferences of the R/I and M-kind since trivially they do not compete for association with the same interval on the degree scale. In sum, the pair “tanto” and “molto” behave differently from the Slovenian “precej” and “veliko” because they do not share the same semantic makeup. This difference sets the perfect ground for a study examining whether cross-linguistic variation potentially feeds cross-linguistic influence, i.e. whether it is possible that bilingual speakers of Slovenian and Italian erroneously treat the pair of m-words in their L2 language in the way they treat the respective pair of m-words in their native language.

Apart from the conventional dominant vs. non-dominant language hypothesis (Kupisch, 2012; Meisel, 2007; Yip & Matthews, 2000), which applies to BLA, L2 acquisition, and heritage language acquisition, suggesting that transfer is more likely from the dominant language to the non-dominant language due to the influence of input on linguistic performance, the literature on cross-linguistic influence presents additional hypotheses, particularly in relation to learnability. These hypotheses include: (1) the partial overlap based on the subset–superset relation between respective grammars (Hulk & Mueller, 2000; Serratrice et al., 2004, 2009; Ionin & Montrul, 2010, a.o.), and (2) the economy hypothesis (Serratrice et al., 2009). While it is unlikely that the L2 language would impact the L1 language in the context of L2 acquisition, factors other than incomplete acquisition may also play a role in creating a suitable context for transfer. Consequently, not every instance of cross-linguistic variation and lower language proficiency is equally prone to triggering negative transfer. Therefore, we briefly explore the latter two hypotheses in relation to the L2 acquisition of Slovenian m-words.

The *subset–superset hypothesis* is based on a learnability model in which when a learner entertains more than one grammatical hypothesis that stand in a subset-superset relation, she is likely to choose the most restricted hypothesis since it is compatible with all types of grammars (Manzini & Wexler, 1987; Wexler & Manzini, 1987). This suggests that the likelihood of the potential transfer in bilingual or L2 acquisition is determined by the relationship between the two languages' grammatical structures. Specifically, if language X has a more restricted set of rules than language Y, and these rules stand in a subset-superset relation, the acquisition of Y as L2 might be associated with negative transfer from X to Y. Considering our case study of m-words, we might extend this hypothesis to the semantics-pragmatics interface, defining Italian as the subset language and Slovenian as the superset language, since the latter presents a more complex interpretative pattern of pragmatic strengthening (Stateva & Stepanov, 2017). According to the subset–superset hypothesis we predicted that Italian (the language in which “molto” and “tanto” are not subjected to enrichment with a stereotypical and non-stereotypical inference, respectively) would influence the L2 acquisition of Slovenian (the language in which “precej” and “veliko” behaved differently when presented in the same context). As a consequence, Italian-Slovenian bilinguals should be willing to treat “precej” and “veliko” similarly compared to Italian monolinguals.

The *economy hypothesis* has been originally based on the evaluation of grammatical options at the syntax-semantics interface. We aim to expand the understanding of economy considerations as a tool for evaluating interpretative proposals at the semantics-pragmatics interface, where potential cross-linguistic influence could be influenced by cognitive factors such as processing efficiency and memory capacity linked to pragmatic enrichment. According to this hypothesis, transfer will occur in whichever direction requires less cognitive effort. Ample evidence suggests that pragmatic interpretations, which involve strengthening semantic meanings with implicatures, come at a cost (cf. Bott & Noveck, 2004). Considering that the Slovenian counterparts of "many" are distinguished by complementing their otherwise equivalent semantic meaning with respective implicatures, while the Italian paradigm does not require the pragmatic extra step in interpretation, one can argue that within the economy hypothesis, the direction of transfer is once again from Italian to Slovenian, if Slovenian is acquired as an L2. If Italian is acquired as L2 by L1 Slovenian speakers, however, one would predict no negative transfer given the economy consideration.

In sum, the two transfer hypotheses are not mutually exclusive and in this case the subset-superset hypothesis and the economy hypothesis lead to the same predictions.

## Our Study

We ask whether L2 acquisition of the vague quantifiers that are equivalent to the English amount quantifier “many” are subject to transfer effects. In other words, we would like to find out if cross-linguistic influence is possible at the semantics-pragmatics interface. In view of the discussion on potential factors triggering negative transfer, we conclude that effects of transfer can be observed from Italian into Slovenian but not vice versa. Therefore, we choose to examine Slovenian, acquired as L2. Both the subset-superset hypothesis and the economy hypothesis make the prediction that Italian learners of Slovenian as L2 are susceptible to transfer and, unlike L1 speakers of Slovenian, they will fail to distinguish between “precej” and “veliko”.

## Methods

### Materials

**Fig. 1:**
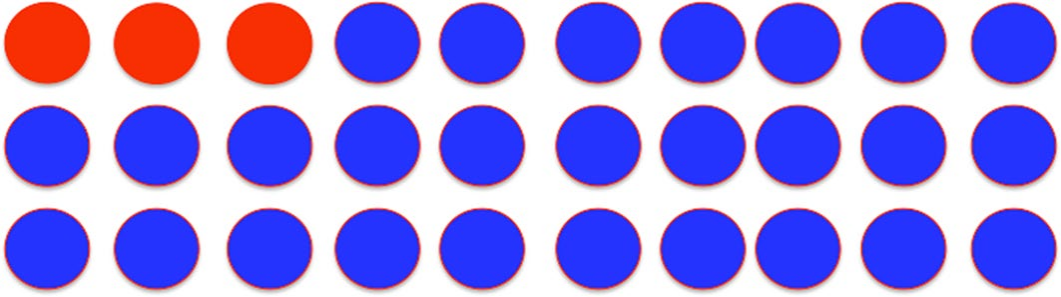
A representative example of an experimental stimulus. The number of blue (here, N =  27) and red (here, N = 3) dots has been manipulated

The experiment is a sentence-picture verification task, and it has been developed by Stateva and Stepanov (2017; *Version III* of the experiments). Participants were first presented with a block of thirty – blue or red – round dots (approx. 1 cm in diameter), like in Fig. 1; the dots were positioned in three rows with 10 dots in each row. The number of red dots has been manipulated and it randomly varied from one to twenty-nine (out of thirty). Each block of dots was positioned in the center of the computer screen and was accompanied below by a set of four sentences. Participants had to evaluate how well a given sentence describes a respective visual context, using a Likert scale indicating appropriateness from 1 (very inappropriate) to 5 (very appropriate). They pressed a radio button. Sentences were in Slovenian and were of the form “QUANTIFIER dots are red”. For each block participants were presented with four sentences, two targets and two fillers.

The two target sentences presented “precej” (1) and “veliko” (2) *m*-words.

**Table:** 

(1)	Precej	točk	je	rdečih
	m-precej	dots.GEN	is	red.GEN
	‘Many dots are red.’			

**Table:** 

(2)	Veliko	točk	je	rdečih
	m-veliko	dots.GEN	is	red.GEN
	‘Many dots are red.’			

Filler sentences had the same structure but the QUANTIFIER could be: (i) *polovica* ‘half’, (ii) *nekaj* ‘a certain amount’, (iii) *vsaj N* ‘at least N’ or (iv) *največ N* ‘at most N’. In (iii) and (iv) N was a natural number that could range from 10 to 27. These fillers varied in equal proportions, one alternation was between (i) and (ii) and another alternation was between (iii) and (iv).

At the end of the experiment, participants had to fill out a test of general language proficiency in Slovenian language: the C-test. First developed by Klein-Braley and Raatz (1984), the test has been used in L2 research as a measure of vocabulary measurement and language proficiency (Karimi, 2011; Singleton & Little, 1991). It consists of texts where the second half of every second word is deleted and participants have to fill it in with the appropriate word-ending. We used three texts developed by a Slovenian native speaker (Vaupotič, 2021).

## Participants

Fifty-two participants took part in our experiment, divided into two groups: 29 Slovenian monolingual speakers (*M*_*age*_ = 34.76*; SD* = 7.37) and 23 Italian speakers with Slovenian as a second language (*M*_*age*_ = 33*; SD* = 7.62). 18 Slovenian monolinguals have been removed for being older than 45 (*N* = 14), for being bilinguals (*N* = 2), or for having scored less than 80% on control items (*N* = 2); 10 Italian bilinguals have been removed for being older than 45 (*N* = 2), for being Slovenian (*N* = 2), for having scored less than 80% on control items (*N* = 3) and for having scored less than 35% at the c-test (*N* = 3).

All participants were recruited via e-mails and social networking forums, and they reported normal or corrected to normal vision and no history of color blindness. We analyzed both groups’ c-test scores. The monolingual Slovenian group scored 91.59% (*SD* = 3.79) while the L2 learners’ group scored 67.7% (*SD* = 21.95).

## Procedure

The experiment was administered online via Ibex farm software (by Alex Drummond, http://spellout.net/ibexfarm/). Participants volunteered and were not compensated for their participation. The first part of the experiments consisted of a questionnaire on their linguistic story, following they run the main experimental session and, finally, fill in the c-test. Items and sentences of the main experimental sessions were pseudo-randomized so that participants could not be sequentially presented with two items with consecutive numbers of dots (e.g., 3 and 4). The three stories of the c-test were presented in random order.

## Results

For the purposes of the analysis, we concentrated on the subset of collected data points that pertain only to our targets “veliko” and “precej”, similar to Stateva and Stepanov (2017). In Table 1 we indicate means and standard deviations (SD) for “veliko” and “precej” in the two groups.

**Table 1:** Mean and SD of “Veliko” and “Precej” in the two groups

Group	Veliko		Precej	
	Mean	SD	Mean	SD
L1	3.04	1.6	2.94	1.5
L2	3.02	1.5	3.01	1.4

We analyzed data with linear mixed-effects models (LMMs) using the lme4 package in R (Bates et al., 2015), considering the acceptability ratings as our dependent variables and participant as random effects. The full model was specified as: Answer_proportion ~ N_Dots * Condition * Group * c_test_percent + (1|ID). The fixed effects in the model included Number of Dots (*N_Dots*), a continuous variable representing the number of dots presented in the experimental stimuli; Condition (*Condition*), a categorical variable with levels corresponding to the quantifiers "veliko" and "precej"; Group (*Group*), a categorical variable indicating whether participants were L1 Slovenian speakers or Italian-Slovenian bilinguals; and C-test Percentage (*c_test_percent*), a continuous variable representing the participants’ scores on the C-test, used as a measure of their general language proficiency in Slovenian. The random effect included a random intercept for each participant (*ID*) to account for the variability across individual participants. The model selection was performed by progressively adding elements (Number of Dots, Condition, Group, and the C-test scores) to the simplest model and comparing the models via Analysis of Variance (ANOVA). According to the Likelihood Ratio Test, the model that best fits the data included all predictors and their interactions (*χ*^*2*^(7) = 19.34; *p* = 0.007). Overall, these results are compatible with the idea that the two groups of speakers give different acceptability scores to “precej” and “veliko” based on the number of dots, with a role of linguistic competence.

To better understand this complex interaction and to focus on the higher part of the scale in which we expect differences among groups of speakers, we decided to conduct a second analysis on the scores given to numbers of dots higher than 25. This kind of analysis is interesting, as stated in Stateva and Stepanov (2017), due to considerations about the nature of the m-words as markers of numerical proportions; specifically, we opted for this high value to be sufficiently confident that it’s a numerical range in which the use of *many* is licensed. Again, we analyzed data with LMMs considering the acceptability ratings (for Number of Dots higher than 25) as our dependent variables and participant as random effects. The model selection was then performed by progressively adding elements (Number of Dots, Condition, Group, and the c-test scores) to the simplest model and comparing the nine models via Analysis of Variance (anova() function in R). In this case, according to the Likelihood Ratio Test, the model that best fits the data included the Number of Dots, Condition and Group in interaction (Answer_proportion ~ N_Dots * Condition * Group + (1 | ID); *χ*^2^(11) = 32.157; *p* = 0.0007).

**Fig. 2:**
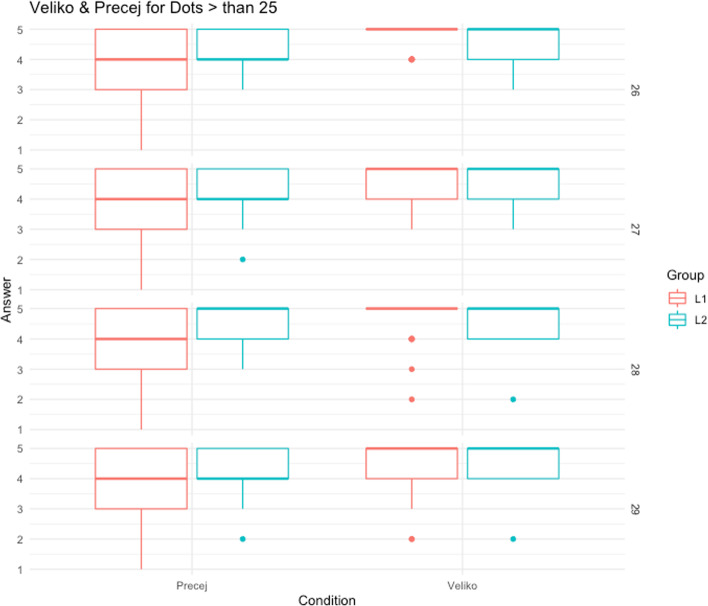
Scores for the condition “Precej” and “Veliko”, in the two groups of Slovenian monolinguals (Group L1, left bars) and Italian-Slovenian bilinguals (Group L2, right bars)

This interaction reveals that the two groups judge differently the two m-word based on the *Number of Dots*. We conducted further Post-hoc pairwise comparisons’ analysis with the “emmeans” package in *r* (Lenth, 2021) and with FDR correction (Benjamini & Hochberg, 1995) on the interaction term “Condition * Group”; results show a significant between-group difference in the score estimations for “precej” (β = -0.116; *SE* = 0.04; *t* = -2.998; *p* = 0.007), with higher scores attributed by L2 speakers, but not for “veliko” (β = 0.008; *SE* = 0.04; *t* = 0.208; *p* = 0.839)*.* The same Post-hoc pairwise comparison with FDR correction on the interaction term “Condition * Group * Number of Dots” shows a significant between-group difference in the score estimations for “precej” uniquely for the highest Number of Dots, that are N = 28 (β = -0.199; *SE* = 0.05; *t* = -3.895; *p* = 0.001) and N = 29 (β = -0.121; *SE* = 0.05; *t* = -2.366; *p* = 0.06). This difference is also visible in Fig. 2.

## Discussion

The results of our study provide evidence that L1 and L2 speakers of Slovenian interpret the m-words “precej” and “veliko” differently based on the number of dots. Specifically, our analysis revealed a significant interaction between Condition, Group, and Number of Dots, indicating that the two groups judge the acceptability of the two m-words differently based on the number of dots. We conducted a second analysis on scores given to numbers of dots higher than 25. Our analysis revealed a significant three-way interaction between Condition, Group, and Number of Dots, suggesting that the effect of linguistic competence on acceptability ratings differs based on the specific m-word and the number of dots presented. Further post-hoc pairwise comparisons were conducted to explore the interaction between Condition and Group, revealing a significant between-group difference in score estimations for “precej”, with higher scores attributed by L2 speakers, but not for “veliko”. Furthermore, the interaction between Condition, Group, and Number of Dots revealed a significant between-group difference in score estimations for “precej” specifically for the highest Number of Dots, which were N = 28 and N = 29. This finding suggests that the effect of language on the interpretation of “precej” is most pronounced when the number of dots is at its highest.

In conclusion, this study aimed to expand research on transfer effects in the pragmatic domain, particularly in the acquisition and use of quantifiers. The way quantities are expressed varies depending on context and language (Katsos et al., 2016; Stateva et al., 2019), and negative transfer theories can be tested in this regard. Our study builds on the previous research by Stateva and Stepanov (2017) on the interpretation of Slovenian m-words, “precej” and “veliko”, and the recent replication of their study in Italian (Mazzaggio & Stateva, 2023). Specifically, in the previous study by Stateva and Stepanov (2017) on the Slovenian language, researchers examined the interpretation of two Slovenian translations of the quantity word "many," namely "veliko" and "precej." The results showed that when the two words were in direct competition, they were not considered equivalent. Instead, it was found that there was a zero tolerance for equal numerical bounds between the two words, leading to a pragmatic strengthening effect under certain contextual conditions. In other words, when "precej" and "veliko" were in competition, "precej" tended to be associated with lower numerical bounds, while "veliko" was associated with higher numerical bounds and more stereotypical interpretations due to an R/I-implicature. A recent study by Mazzaggio and Stateva (2023) failed to replicate these findings in the Italian language. Italian native speakers did not differentiate between "molto" and "tanto" when presented as alternatives, in contrast to the results obtained with Slovenian speakers. These results suggest that the Italian m-words, "molto" and "tanto," have the same numerical bounds and are therefore perceived as interchangeable when used as amount modifiers. This contrast between Slovenian and Italian m-words provided the perfect opportunity to investigate potential cross-linguistic transfer effects in the interpretation of quantifiers.

The present study aimed to examine whether Italian-Slovenian bilinguals exhibit negative transfer. We tested non-fully proficient L2 speakers because cross-linguistic influence is expected in cases of incomplete acquisitions (Polinsky, 2018). The experiment involved two groups of participants who were tested in Slovenian on their interpretation “precej” and “veliko”: Slovenian monolingual speakers and Italian-Slovenian bilinguals. Firstly, we replicated results of Stateva and Stepanov (2017) with the same population (Slovenian monolinguals) since “precej” and “veliko” have not been interpreted in the same way. Secondly, in line with our predictions, Italian-Slovenian bilinguals exhibited a failure to differentiate between the two m-words in Slovenian, and instead displayed a tendency to behave similarly to Italian speakers, which may be due to negative transfer. Notably, we found a substantial difference in the scores attributed to “precej” (but not “veliko”), particularly in cases where the participants evaluated contexts with 28 or 29 dots out of a total of 30. This is precisely where monolingual Slovenians attributed the highest values (as measured by the Likert scale) to *“*veliko” (i.e., for higher number of dots, Slovenian prefer to use “veliko”).

While our premises and previous analyses lead us to favor interpreting our results as an effect of pragmatic negative transfer, it is crucial to consider alternative explanations for our findings. One such alternative is the "lexical alternative hypothesis" (Jarvis & Pavlenko, 2008), based on which L2 speakers might interpret both "precej" and "veliko" as equivalent to the Italian "molto/tanto". This hypothesis suggests that any observed differences are due to lexical transfer at the word level or their lexical semantics, rather than pragmatic transfer. According to this view, L2 speakers may assign the same lexical semantics to "precej" and "veliko" as they do to "molto/tanto", potentially explaining the lack of differentiation observed in their responses. Moreover, our results could be interpreted through the lens of simplified acquisition, a phenomenon well-documented in second language acquisition literature (a.o, Klein & Perdue, 1997). L2 speakers might have acquired a basic semantics of "precej" and "veliko," capturing the general function of many-quantifiers without internalizing the nuanced differences that native speakers understand. This simplified acquisition could result from various factors, including limited exposure to the target language or cognitive constraints in L2 processing.

However, we argue that these alternative hypotheses do not fully capture our results. The "lexical alternative hypothesis" requires a precise semantic definition to be properly evaluated. Previous research supports the hypothesis we follow in this study. Specifically, Stateva and Stepanov (2017) have shown that in the absence of alternatives, "precej" and "veliko" do not differ in terms of their numerical boundaries; they are treated synonymously by both groups of participants. The current article considers previous findings and replicates the "precej/veliko" results of the experiment, in which participants evaluate the meaning of sentences with the m-word in the presence of both alternatives, facilitating sensitivity to the differences between the two interpretations. Since, to our knowledge, no other hypotheses in the literature explain the differences between the two Slovenian m-words, this paper relies on the unique pragmatic strengthening explanation.

The above mentioned debate surely underscore the necessity for further systematic empirical assessment to pinpoint the exact mechanisms behind the observed cross-linguistic influence. This could involve comparing the interpretation of "precej" and "veliko" to other instances of R/I-implicature or M-implicature strengthening, such as typical cases illustrated by Levinson (1987, 2000), Horn (1989) and Krifka's (2007) antonyms.

Overall, our findings suggest that being an L2 learner influences the interpretation of quantifiers in Slovenian, and that L2 speakers may have a different understanding of these words compared to native speakers. The fact that Italian-Slovenian bilinguals failed to differentiate between the two m-words in Slovenian, behaving like in Italian, suggests that negative transfer might have played a role. Bilinguals may rely more on their dominant language knowledge when processing language, which can affect their understanding of quantifiers in the non-dominant language.

These findings have important implications for language acquisition and education. They suggest that bilinguals may need additional support and instruction to fully grasp the nuances of quantifiers in their non-dominant language. Educators and language professionals should be aware of the potential for negative transfer and design language programs that consider the linguistic and cultural differences between languages.

Overall, this study contributes to our understanding of how bilinguals process and interpret quantifiers and highlights the importance of considering the role of cross-linguistic influence in language acquisition and use. Further research is needed to investigate the generalizability of our findings to other language pairs and to explore the cognitive mechanisms underlying transfer effects in the pragmatic domain.

## Conclusion

Our study demonstrates that, not only we replicated the results from Stateva and Stepanov (2017) and thus we support the hypothesis that Slovenian m-words are distinguished pragmatically through a mechanism of strengthening by implicatures but we also identified a new domain of cross-linguistic influence. To the best of our knowledge, this is the first investigation of negative transfer in the domain of L2 acquisition of quantifiers. This study enhances our understanding by highlighting that cross-linguistic influence extends beyond distinct grammar domains. It can be seen as an interface phenomenon encompassing not only the syntax-pragmatics interface (cf. Serratrice et al., 2004) and the syntax-semantics interface (cf. Serratrice et al., 2009), but also the semantics-pragmatics interface. Additionally, we would like to emphasize that our results potentially support both the subset-superset hypothesis and the economy hypothesis.

## Footnotes

1 Alternatively, “many” is defined in a manner similar to the other quantificational determiners as a (characteristic function of) a relation between sets of individuals. For example, in its proportional meaning it is specified as in (i): (i) $\left[\kern-0.15em\left[ {many} \right]\kern-0.15em\right] ^{c} = {\lambda A}.{\lambda B}.\frac{{\left| {{\text{A}} \cap {\text{B}}} \right|}}{{\left| {\text{A}} \right|}}{\text{ n}}_{{\text{c}}}$, for some large n_c_.

2 $$\left[\kern-0.15em\left[ {many} \right]\kern-0.15em\right] = {\lambda d}_{{\text{d}}} .{\lambda I}_{{ < {\text{dt}} > }} .{\text{I}}({\text{d}}).$$

3

- *R-principle*:

Make your contribution necessary, say no more than you must. (Horn, 1984: 13).

- *I-Principle*:

(i) *Speaker’s Maxim: the Maxim of Minimization.* ‘Say as little as necessary’, i.e., produce the minimal linguistic information sufficient to achieve your communicational ends (bearing the Q-principle in mind). (ii) *Recipient’s Corollary: the Enrichment Rule.* Amplify the informational content of the speaker’s utterance, by finding the most SPECIFIC interpretation, up to what you judge to be the speaker’s m-intended point. (Levinson, 1987: 402).

- *M-Principle:*

(i) *Speaker’s Maxim*: Indicate an abnormal, non-stereotypical situation by using marked expressions that contrast with those you would use to describe the corresponding normal stereotypical situation. (ii) *Recipient’s Corollary*: What is said in an abnormal way indicates an abnormal situation, or marked messages indicate marked situations. […] (Levinson, 2000: 136).

4 Another one, “Parecchio”, which is less common, will not be taken into consideration. When searching the LIP corpus (De Mauro et al., 1993) for "parecchio," it received a score of 33 for adjectival usage, 6 for pronominal usage, and 3 for adverbial usage.

5 For more detailed description of the meanings of “molto” and “tanto” as well as their differences in distribution we refer the reader to Mazzaggio and Stateva (2023).
